# Accuracy of visual estimation of shoulder abduction by expert physiotherapists: a comparative study using 3D motion capture

**DOI:** 10.1186/s12891-026-10073-y

**Published:** 2026-06-16

**Authors:** Martin Kenner, Phillip Thies, Annika Schwarz

**Affiliations:** Faculty of Social Sciences - Applied Therapeutic Sciences, City University of Applied Sciences Bremen, Bremen, Germany

**Keywords:** Shoulder joint, 3D motion capture, Range of motion, Visual estimation, Measurement agreement, Bland–Altman analysis

## Abstract

**Background:**

Accurate assessment of shoulder range of motion is essential for clinical decision-making in physiotherapy. While visual estimation of joint angles is commonly used in practice, its agreement with objective reference measurements under clinically relevant conditions remains unclear. This study aimed to assess the agreement between video-based expert visual estimation of shoulder abduction and three-dimensional (3D) motion capture as a reference standard.

**Methods:**

A cross-sectional method comparison study was conducted. Shoulder abduction movements of four healthy participants were recorded using a 3D motion capture system. Twenty-four video recordings were presented to experienced physiotherapists (≥ 10 years of clinical experience), who visually estimated joint angles via an online questionnaire. Differences between estimated and reference values were analysed using descriptive statistics, Bland–Altman analysis, and cross-classified mixed-effects modelling to account for repeated measurements across raters and video stimuli.

**Results:**

A total of 923 paired observations from 33 experts were analysed. Visual estimation showed a consistent tendency toward overestimation, with a mean difference of 14.7° (SD 11.6°) and limits of agreement ranging from − 8.0° to 37.5°. A cross-classified mixed-effects model estimated a mean bias of 14.5° (95% CI 11.8° to 17.3°), confirming systematic overestimation. Most estimates (89.9%) exceeded the reference value. Variability was substantial, with 27.3% of variance attributable to differences between video stimuli and 10.5% to differences between raters.

**Conclusion:**

Visual estimation of shoulder abduction angles by experienced physiotherapists showed limited agreement with a 3D motion capture reference and a consistent tendency toward overestimation. Given the observed variability, visual estimation alone may be insufficient for accurately detecting small changes in joint angle. The use of objective measurement tools may support more reliable assessment in clinical practice.

**Supplementary Information:**

The online version contains supplementary material available at 10.1186/s12891-026-10073-y.

## Introduction

Shoulder disorders are common in physiotherapy practice and represent a substantial burden in musculoskeletal care. Globally, shoulder pain has a median prevalence of approximately 16% in the general population, with considerable variation across settings [1]. In routine care, shoulder conditions account for a relevant proportion of physiotherapy cases, as reflected in registry-based data [2]. The condition of increased shoulder pain is significantly associated with reduced shoulder range of motion and increased physical disability [3]. Given their impact on upper limb function, shoulder disorders can be associated with limitations in daily activities and participation. Adequate shoulder abduction is essential for performing activities of daily living, with approximately 125° required for functional tasks [4]. In healthy individuals, maximal shoulder abduction typically exceeds this threshold, averaging around 150° [5]. In clinical practice, accurate assessment of joint angles is therefore important for diagnosis, treatment planning, and monitoring of rehabilitation outcomes [6]. Various measurement tools, such as goniometers, inclinometers, and validated smartphone applications, are available and have demonstrated acceptable measurement properties [7, 8]. However, visual estimation of joint angles is still commonly used in clinical practice, particularly in situations where time or resources limit the use of measurement instruments [9]. Previous studies have reported deviations between visually estimated and instrument-based measurements of joint angles. For example, Russo et al. [10] found a mean absolute difference of 6.5° (SD = 5.3°) between visual estimations and reference values. Similarly, Lang & Milosavljevic [11] demonstrated significant discrepancies between visual estimates by physiotherapy students and computer-based measurements. Even relatively small deviations in joint angle measurement may be clinically relevant. For example, the minimal clinically important difference (MCID) for shoulder abduction following arthroplasty has been reported to be approximately 7° [12], indicating that differences between visually estimated and reference values may overlap with clinically meaningful change, particularly when monitoring rehabilitation progress.

However, existing evidence includes studies based on passive movement conditions or non-expert raters [10, 11], which may not reflect typical clinical assessment scenarios. Consequently, the accuracy of visual estimation under clinically relevant conditions, particularly when performed by experienced physiotherapists during active movement, remains unclear. Therefore, the aim of this study was to assess the agreement between video-based visual estimation of shoulder abduction angles by experienced physiotherapists and a three-dimensional motion capture reference standard. 

## Methods

### Study design and participants

A cross-sectional method comparison study was conducted to assess the agreement between visual estimation of shoulder abduction angles and reference measurements obtained using the Vicon Nexus system. The study followed the PIRD (Population, Index test, Reference standard, Diagnostic target) framework [13], with healthy individuals as the population, visual estimation as the index test, 3D motion capture measurements as the reference standard, and shoulder abduction angle as the target construct. The study was preregistered prior to data collection (DOI: 10.17605/OSF.IO/NFHX6) to ensure good scientific practice [14].

The sample size was planned a priori using MedCalc software (version 22.018, MedCalc Software Ltd., Ostend, Belgium) based on a Bland–Altman approach for paired observations and the recommendations by Lu et al.[15] . The alpha level was set at 0.05 and a power of 80% was assumed. Parameters were derived from Russo et al.[10] with an expected mean difference of 6.5° (SD = 5.3°), resulting in a required sample size of at least 525 paired measurements. As sample size planning for cross-classified mixed-effects models typically requires simulation-based approaches, a Bland–Altman-based calculation was used as a pragmatic approximation to ensure a sufficient number of paired observations. To generate an adequate number of observations for the planned analysis, 24 shoulder abduction videos were recorded and implemented in an online questionnaire.

Movement recordings were obtained from four healthy participants recruited using a convenience sampling approach. Participants were required to be free of upper limb pathology. The participants varied in age, sex, and body composition (ectomorphic, mesomorphic, and endomorphic), providing heterogeneity across the recorded videos. Exclusion criteria included upper limb deformities, identifiable tattoos in the filmed area, and pain during movement.

Experts were defined as physiotherapists with at least ten years of professional experience in musculoskeletal care. This threshold was chosen to reflect an advanced level of clinical expertise, consistent with models of skill acquisition in healthcare [16, 17]. Recruitment was conducted through professional networks, social media platforms (Instagram, Facebook, LinkedIn), and the German Association for Manual Therapy (Maitland^®^ Concept). No additional demographic variables were collected.

All procedures involving human participants were performed in accordance with the ethical standards of the institutional research committee and with the Declaration of Helsinki. Informed consent was obtained from all participants prior to participation. Data were pseudonymised (video models) or anonymised (experts). 

### Experimental setup

The Vicon motion capture system (Vicon Motion Systems Ltd., Oxford, UK) was used to record precise three-dimensional movement data during shoulder abduction. This system is widely regarded as a reference standard and is particularly suited for controlled experimental setups [18–20]. It employs high-resolution infrared cameras to detect passively reflective markers placed on the body, enabling reconstruction of a three-dimensional kinematic model using Vicon Nexus software (version 2.12.0).

**Fig. 1:**
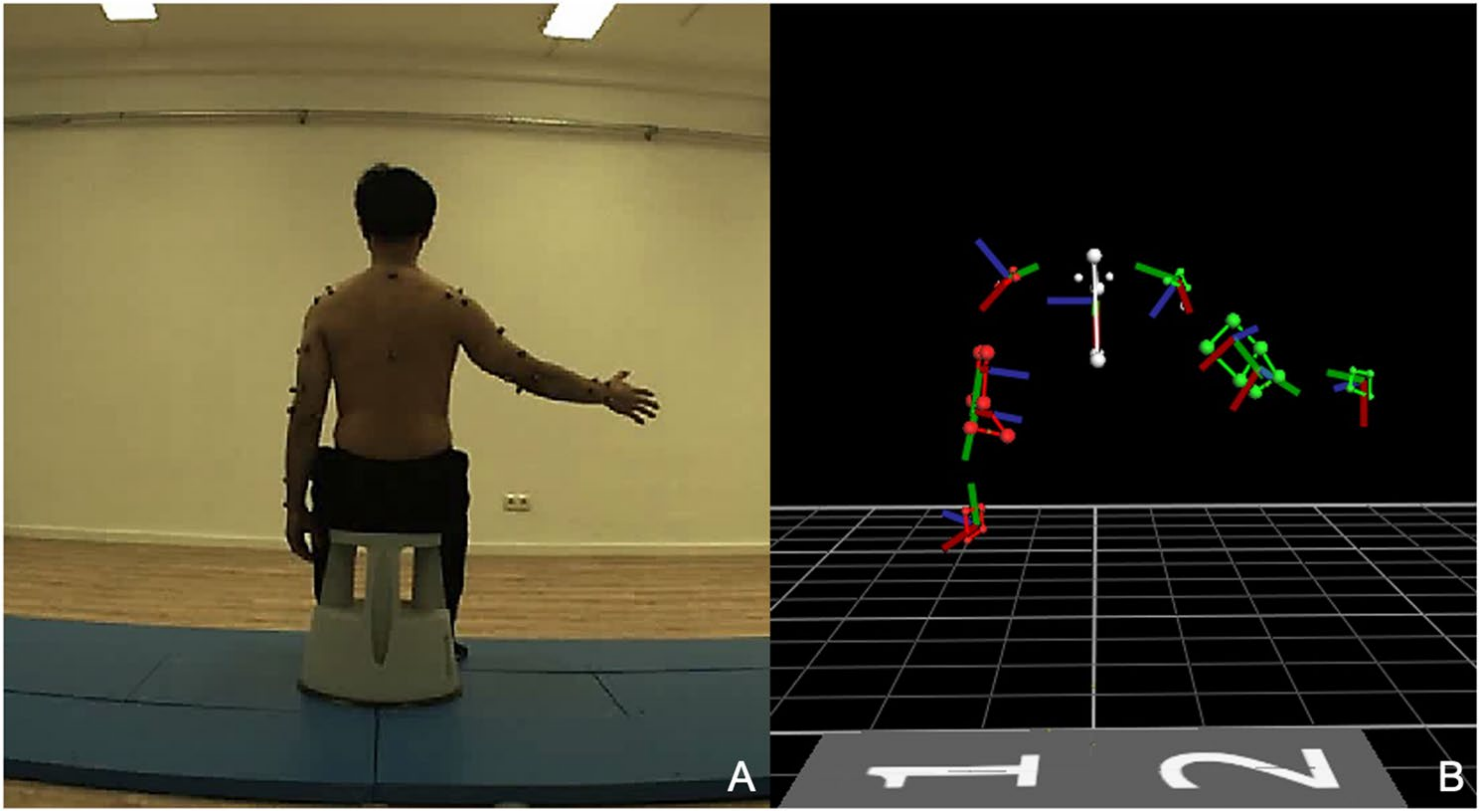
Experimental setup. **A **Video recording of a participant performing shoulder abduction as presented to the raters. **B** Corresponding 3D motion capture reconstruction used as the reference standard

Movements were recorded in the motion laboratory at City University of Applied Sciences Bremen using eight infrared cameras and one digital video camera (Fig. 1). Participants were seated on a 42 cm high seat at a distance of approximately 2.8 m from the video camera, which was positioned at a height of 75 cm. Participants faced away from the camera to ensure anonymity and to replicate a dorsal viewing perspective commonly used in clinical assessment (Fig. 1A). The reference value of shoulder abduction was calculated using the Upper Limb Kinematic Marker Model (ULKM; Fig. 1B), which follows recommendations of the International Society of Biomechanics [21].

## Procedures

### Motion capture recordings

Each of the four participants was recorded individually over two days (15–16 February 2024). Thirty-two reflective markers were placed according to anatomical reference points defined by the ULKM [22] and secured using skin-friendly adhesive tape. The system was calibrated for each participant before data collection. Participants performed cyclical shoulder abduction movements starting from the neutral position. Movements were performed at a moderate speed and interrupted by randomly timed stop signals. During each stop, the arm position was held for approximately three seconds before the movement resumed. Each participant performed three repetitions for both the right and left arm, resulting in six recordings per participant and a total of 24 recordings. Recordings were reviewed and repeated if necessary; one recording required repetition due to marker loss. Each recording session lasted approximately 75 min.

### Visual estimation procedure

The index test consisted of expert physiotherapists visually estimating shoulder abduction angles based on recorded videos. Video recordings were embedded in an online questionnaire created using LimeSurvey (version 6.4.2) and distributed to participants. Figure 1A ilustrates a representative screenshot of the video presentation used for visual estimation. Participation required confirmation of at least ten years of professional experience in physiotherapy; participants not meeting this criterion were excluded automatically. Each video showed one shoulder abduction movement, and experts were instructed to estimate the angle during the approximately three-second holding phase. Each video was presented once, and experts were asked to provide their estimate in degrees. The questionnaire included all 24 recorded videos and remained open for three weeks (February 19 to March 11, 2024). Data were stored securely on German LimeSurvey servers (LimeSurvey GmbH, 2024). The questionnaire was specifically developed for this study and is provided as Supplementary File 1.

### Statistical analysis

Estimated and reference values were first compiled into a Microsoft Excel spreadsheet (version 2108). Statistical analysis was then conducted using STATA (version 17BE, StataCorp LLC). Differences between visual estimates and reference values were calculated (diff = estimate − reference), with positive values indicating overestimation. Absolute differences were additionally computed to quantify overall deviation irrespective of direction.

Descriptive statistics (mean, standard deviation, median, interquartile range, minimum and maximum) were calculated for the differences between visual estimates and reference values, as well as for the absolute differences. The direction of deviations (overestimation, underestimation, exact agreement) was summarised using frequencies and percentages.

Bland–Altman analysis was conducted to provide a descriptive assessment of agreement between methods. Mean difference and standard deviation of the differences were calculated, and limits of agreement (LoA = mean ± 1.96 SD) were derived [23]. Given the presence of repeated measurements, Bland–Altman results were interpreted descriptively rather than inferentially [24, 25].

As multiple ratings were provided by the same raters across the same video stimuli, the data had a cross-classified structure with non-independent observations [26, 27]. To account for this, the primary analysis was conducted using a cross-classified linear mixed-effects model [27–29]. The difference between estimated and reference values served as the dependent variable, with the intercept representing the mean systematic bias. Random intercepts were specified for both rater and video to account for clustering at both levels. Parameters were estimated using restricted maximum likelihood (REML), which provides less biased variance estimates compared to maximum likelihood estimation in mixed models [29, 30]. To explore potential proportional bias, an additional cross-classified mixed-effects model including the reference angle as a fixed effect was performed.

A sensitivity analysis was performed using a mixed-effects model including video as the only random effect to assess the robustness of the estimated bias under alternative model specifications.

All available angle estimations were included in the final analysis. Partial questionnaire responses were retained if angle values had been provided. No data imputation procedures were applied.

## Results

**Fig. 2:**
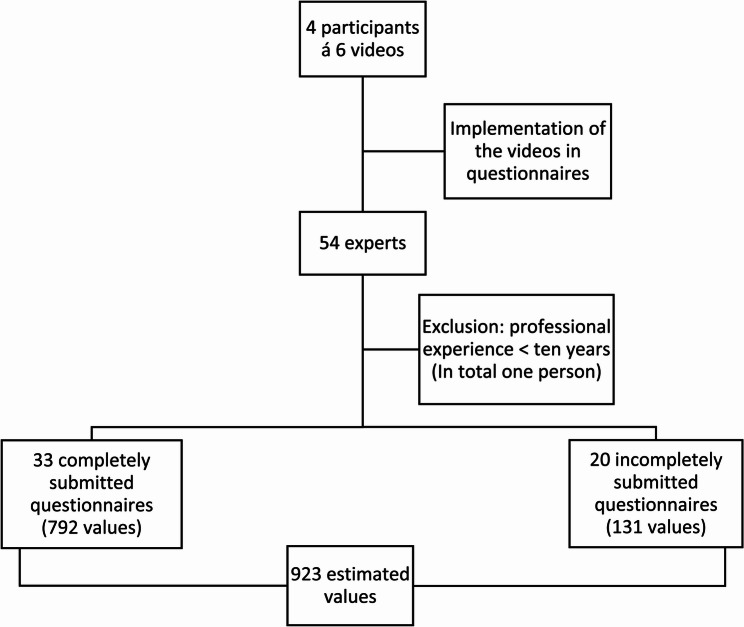
Flowchart of data collection

Of 54 experts who accessed the questionnaire, 33 completed it fully. One participant was excluded due to less than 10 years of professional experience. In total, 923 joint angle estimates (Fig. 2) were collected. Reference values ranged from 16° to 142°. All available angle estimations were included in the final analysis, including those from partially completed questionnaires where angle values were provided. No data imputation was performed.

The maximum underestimation was 23°, while the maximum overestimation reached 62°. Out of the 923 estimates, 34 (3.7%) accurately matched the reference value (Table 1). A total of 59 estimates (6.4%) underestimated the angle, while 830 estimates (89.9%) overestimated it.

**Table 1:** Percentages of overestimation and underestimation

	Exact agreement	Underestimated	Overestimated
Total (*n* = 923)	3.7% (34 Values)	6.4% (59 Values)	89.9% (830 Values)
Left Side (*n* = 471)	1.9% (9 Values)	4.7% (22 Values)	93.4% (440 Values)
Right Side (*n* = 452)	5.5% (25 Values)	8.2% (37 Values)	86.3% (390 Values)

*n * Number of paired values

The observed mean difference between estimated and reference values was 14.7° (SD = 11.6°). Absolute differences averaged 15.5° (SD = 10.5°). Further agreement measures and mixed-effects model results are presented in Table 2.

**Table 2:** Summary of agreement measures and mixed-effects model results

Outcome measure	Estimate
Mean difference (°), mean ± SD	14.7 ± 11.6
Absolute difference (°), mean ± SD	15.5 ± 10.5
Lower limit of agreement (°)	−8.0
Upper limit of agreement (°)	37.5
Mixed-effects model (bias, °), estimate [95% CI]	14.5 [11.8; 17.3]
Variance components (random effects)	
Video-level variance (σ²), % of total variance	37.3 (27.3%)
Rater-level variance (σ²), % of total variance	14.3 (10.5%)
Residual variance (σ²), % of total variance	84.9 (62.2%)

Mean difference and absolute difference are reported as mean ± standard deviation (SD). Limits of agreement were calculated as mean ± 1.96 SD. The mixed-effects model estimate represents the mean systematic bias with 95% confidence interval (CI). Variance components are reported as variance (σ²) and percentage of total variance

An additional analysis assessing proportional bias showed no significant association between the reference angle and the difference between visual estimation and the reference measurement (β = 0.054, 95% CI −0.026 to 0.134, p = 0.186).

To further explore variability across stimuli, Table 3 presents descriptive statistics for each video stimulus, listed in the order in which they were presented in the questionnaire. Video 13 showed the smallest mean difference (0.8°, SD = 9.2), whereas video 21 showed the largest mean difference (24.3°, SD = 15.1). The lowest variability was observed for video 9 (SD = 2.0), while the highest variability was found for video 4 (SD = 16.5).

**Table 3:** Descriptive statistics for each video stimulus

Video ID	*N*	Reference value (°)	Mean estimate (°)	Mean difference (°)	Standard deviation (°)
01	53	90	112.7	22.7	10.9
02	52	95	106.3	11.3	11.8
03	50	73	85.6	12.6	6.8
04	48	128	145.4	17.4	16.5
05	46	129	152.7	23.7	16.1
06	43	65	80.8	15.8	5.7
07	41	87	96.1	9.1	10.5
08	38	133	155.5	22.5	15.9
09	38	78	89.0	11.0	2.0
10	37	88	103.7	15.7	12.7
11	37	142	160.4	18.4	12.8
12	37	16	32.5	16.5	7.4
13	36	101	101.8	0.8	9.2
14	36	80	86.5	6.5	4.2
15	34	79	87.1	8.1	4.8
16	33	64	86.2	22.2	3.7
17	33	30	38.5	8.5	5.8
18	33	53	74.9	21.9	5.8
19	33	45	60.2	15.2	9.7
20	33	80	88.8	8.8	4.7
21	33	126	150.3	24.3	15.1
22	33	73	86.5	13.5	4.7
23	33	81	89.2	8.2	5.2
24	33	80	93.9	13.9	7.0

*N  *Number of paired values, *ID*  Identification Number

*° angular values expressed in degrees*

### Analysis of mean bias and limits of agreement

**Fig. 3:**
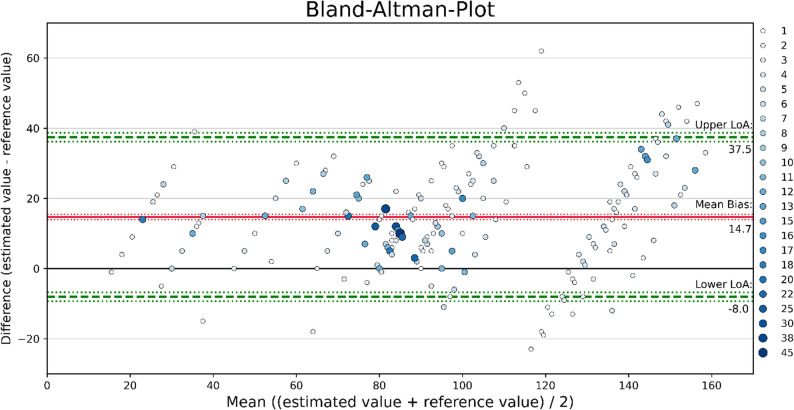
Bland-Altman-Plot of estimated versus reference values. The solid line represents the mean difference (Mean Bias), and the dashed lines indicate the limits of agreement (LoA); point size and colour intensity reflect the number of overlapping observations.

The Bland–Altman analysis yielded limits of agreement ranging from − 8.0° to 37.5°. Figure 3 illustrates the distribution of differences across the range of measured values. The distribution of differences is predominantly above zero, indicating a tendency toward overestimation of shoulder abduction angles.

To account for the clustered structure of the data, inferential analysis was performed using a cross-classified mixed-effects model. The model estimated a mean difference of 14.5° (95% CI: 11.8° to 17.3°), indicating a consistent overestimation. Variance decomposition revealed that 27.3% of the total variance was attributable to differences between videos, 10.5% to differences between raters, and 62.2% to residual variability.

**Fig. 4:**
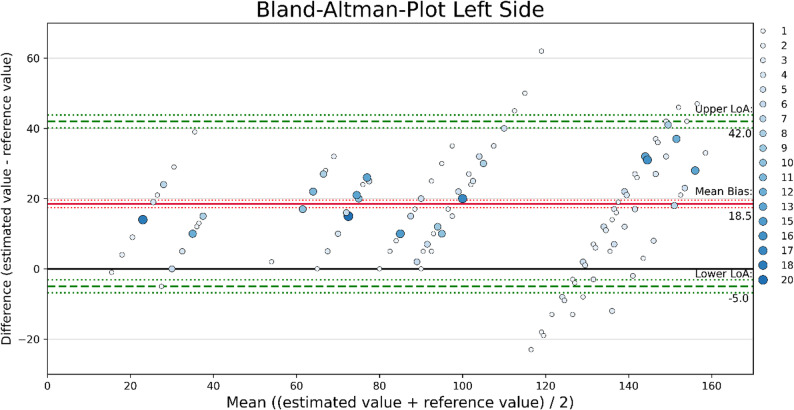
Bland-Altman-Plot for the left side. The solid line represents the mean difference (Mean Bias), and the dashed lines indicate the limits of agreement (LoA); point size and colour intensity reflect the number of overlapping observations

**Fig. 5:**
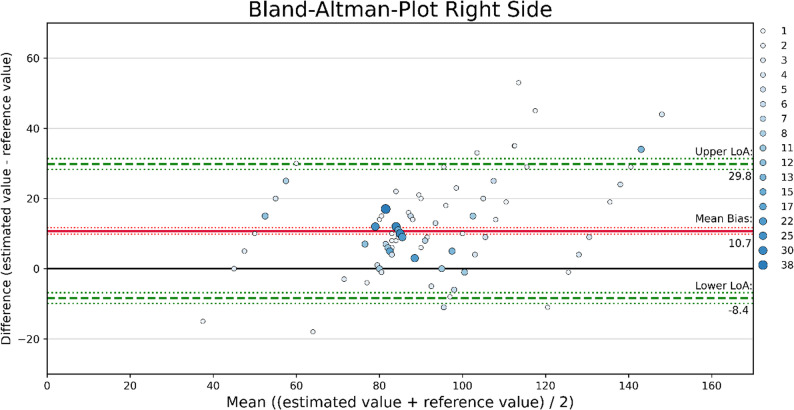
Bland-Altman-Plot for the right side. The solid line represents the mean difference (Mean Bias), and the dashed lines indicate the limits of agreement (LoA); point size and colour intensity reflect the number of overlapping observations

Separate descriptive Bland–Altman analyses were conducted for the left and right side. For the left side (Fig. 4), the mean difference was 18.5°, with limits of agreement from − 5.0° to 42.0°. For the right side (Fig. 5), the mean difference was 10.7°, with limits of agreement from − 8.4° to 29.8°. These results indicate a tendency toward stronger overestimation on the left side compared to the right.

## Discussion

This study evaluated the agreement between expert visual estimation of shoulder abduction angles and a validated 3D motion capture reference. The results indicate a consistent overestimation accompanied by wide limits of agreement, reflecting substantial variability between estimated and reference values. Findings from the cross-classified mixed-effects model were consistent with this pattern, supporting the presence of a systematic bias. Taken together, these results suggest that visual estimation and instrument-based measurement cannot be used interchangeably.

The predominance of overestimation observed in this study suggests the presence of a systematic tendency in visual angle assessment. One possible explanation is the absence of external reference points, which may limit the accuracy of visual judgments. In addition, estimation under active movement conditions may introduce greater perceptual complexity compared to static or instrument-assisted measurements, potentially contributing to increased variability.

Previous research has shown that even trained observers may have limited accuracy when estimating joint angles without the use of measurement instruments [10, 11]. The magnitude and direction of deviations observed in the present study are consistent with these findings, indicating that visual estimation, even among experienced clinicians, is associated with systematic differences compared to reference-based measurements.

Further comparison with previous findings highlights the influence of study design on estimation accuracy. Russo et al. [10] reported a lower mean estimation error for shoulder abduction (6.5° ± 5.3°) under controlled conditions using cadaver specimens and passive joint positioning. In contrast, the present study involved active movements performed in a seated position, with participants maintaining the target position for several seconds to better approximate clinical assessment conditions. These methodological differences may partly explain the larger deviations observed in the current study, as estimation under active conditions may be subject to greater variability than under passive, highly controlled settings. In addition, no evidence of proportional bias was observed, suggesting that the magnitude of estimation error did not systematically increase at higher shoulder abduction angles.

The clinical relevance of these findings can be considered in relation to thresholds of clinically meaningful change. For example, the minimal clinically important difference (MCID) for shoulder abduction following arthroplasty has been reported to be approximately 7° [12]. In contrast, the systematic overestimation observed in this study, reflected by a mean bias of approximately 14.5° and wide limits of agreement, was substantially larger. This indicates that visual estimation may have limited sensitivity for detecting small but clinically relevant changes in joint angle. Consequently, reliance on visual estimation alone may hinder the interpretation of subtle changes over time in clinical practice.

Alternative measurement approaches may help to address the variability observed in visual estimation. Commonly used tools such as goniometers and inclinometers provide more standardized assessments of joint angles. In addition, smartphone-based applications have been investigated as accessible options for joint angle measurement, with reported errors ranging from − 0.41° to 6.8° when compared to goniometric measurements [31–33]. At the same time, Patient-Reported Outcome Measures (PROMs) are increasingly used in physiotherapy and may complement instrument-based assessments (e.g. goniometric or inclinometric measurements) rather than replace them. As range-of-motion measures are often associated with functional outcomes [4], combining subjective and objective information may support more comprehensive clinical decision-making [4, 34, 35]. In addition, the observed tendency toward overestimation may have practical implications for clinical training. Raising awareness of this systematic bias could support more accurate visual assessments and improve the interpretation of joint angle changes in routine practice. 

## Strength and limitations

This study has several important strengths. With 923 paired observations, it represents one of the largest datasets investigating visual estimation of a specific joint movement. The use of a cross-classified mixed-effects model allowed appropriate handling of the non-independent data structure by accounting for clustering at both the rater and video level, thereby providing more robust estimates of systematic bias. In addition, the inclusion of active, functionally relevant movements enhances the clinical applicability of the findings compared to previous studies conducted under passive or highly controlled conditions. Finally, the recruitment of experienced clinicians from a wide geographical area supports the external relevance of the results.

Despite these strengths, several limitations should be considered. First, the analysis was based on a small number of movement recordings from four healthy participants, which may limit generalisability across different body types, movement patterns, and clinical populations. Second, individual leftacteristics such as gender, body weight, and height were not analysed, although these factors may influence visual estimation accuracy. Third, the study population consisted exclusively of experienced physiotherapists, which may limit transferability to less experienced clinicians. In addition, visual estimation was performed using two-dimensional video recordings within an online questionnaire rather than in-person assessment. This may reduce ecological validity compared to clinical settings, where three-dimensional perception and direct interaction are available. Variations in display size and resolution across participants’ devices could not be controlled, although the standardized presentation of the questionnaire helped to reduce variability. As each video was rated only once, intra-rater consistency could not be assessed.

## Conclusion

Visual estimation of shoulder abduction angles showed a consistent tendency toward overestimation and considerable variability compared to a reference-based measurement. These findings suggest limited agreement between visual and instrument-based assessments and indicate that visual estimation may have restricted sensitivity for detecting small changes in joint angle.

In clinical contexts where joint angle measurements are used to monitor change over time, this level of variability may complicate the interpretation and documentation of observed differences. This is particularly relevant when small improvements are expected, as measurement variability may be of a similar magnitude as clinically meaningful change. In such contexts, more standardized measurement approaches may support more consistent interpretation.

## Supplementary Information

Supplementary Material 1.

## Data Availability

The datasets generated for this study are available on request to the corresponding author.

## Abbreviations

CI: Confidence Interval
DOI: Digital Object Identifier
MCID: Minimal Clinically Important Difference
OSF: Open Science Framework
PIRD: Population, Index test, Reference standard, Diagnostic target
RM: Reflective Markers
ROM: Range of Motion
SD: Standard Deviation
ULKM: Upper Limb Kinematic Model
