# Supplementary material for: Accuracy of visual estimation of shoulder abduction by expert physiotherapists: a comparative study using 3D motion capture
Source: BMC Musculoskelet Disord. 2026 Jun 16;27:514. doi: 10.1186/s12891-026-10073-y (PMC13270789; doi:10.1186/s12891-026-10073-y)
Supplement: Supplementary file 1 — Supplementary Material 1. [file 12891_2026_10073_MOESM1_ESM.pdf]

Supplementary File 1

English version of the online questionnaire (LimeSurvey) used in this study.

Question 1 *(Screening)*

Do you have at least ten years of professional experience in physiotherapy?

☐ Yes

☐ No

Note:<br>*Participants selecting "No" were automatically excluded from further participation.*

Questions 2–25 *(Angle Estimation Items*)

Instruction:

Please estimate the joint angle in the holding position in degrees. Please watch the video only once.

Response field:

______ °

Note:<br>*Each item consisted of:*<br>• *One embedded video showing a shoulder abduction movement*<br>• *A numeric input field for entering the estimated joint angle (in degrees)*<br>*The 24 videos were displayed sequentially. Each video corresponded to one estimation task.*<br>*No feedback on accuracy was provided during the questionnaire.*
